# Predicting dengue where surveillance is weak: A case for climate- and search-based machine learning in Somalia

**DOI:** 10.1016/j.nmni.2026.101817

**Published:** 2026-07-22

**Authors:** Mohamed Mahdi Hashi, Abdisamad Abdihakim Aadam, Abdishakur Ahmed Mahad, Abdimalik Dahir Ali, Yusuf Abdullahi Hubow

**Affiliations:** a Faculty of Health Sciences and Tropical Medicine, Somali National University, Mogadishu, Somalia; b Benaadir Research, Consultancy & Evaluation Center, Mogadishu, Somalia; c Center for Health Research and Innovation, Somali National University, Mogadishu, Somalia

**Keywords:** Dengue fever, Machine learning, Climate variability, Disease surveillance, Somalia

Dear editor

Dengue is among the fastest-spreading vector-borne infections, and outbreaks can overwhelm health systems when early warning is absent [1]. Somalia now faces a neglected but increasingly documented dengue burden, with confirmed outbreaks in Banadir and Puntland and historical evidence of circulation dating back decades [2,3]. In such settings, delayed reporting and weak laboratory infrastructure make conventional surveillance too reactive to prevent localized escalation [3,4] (see Fig. 1).

**Fig. 1:**
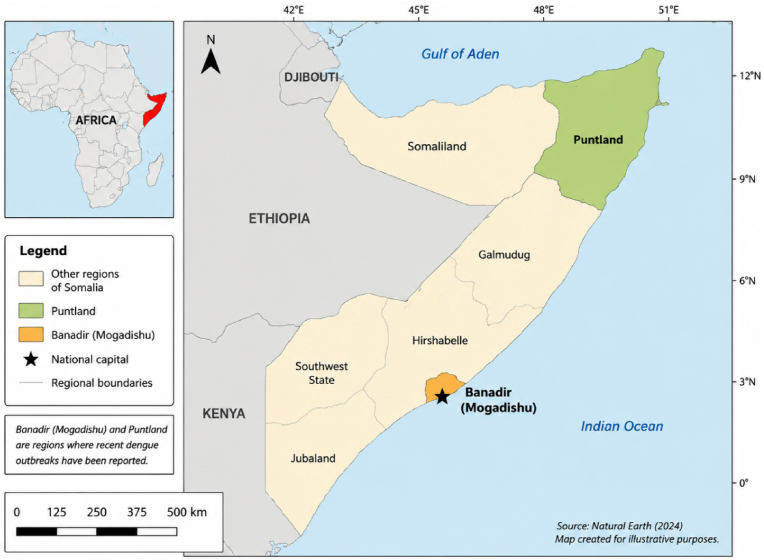
Map of Somalia in the Horn of Africa highlighting Banadir (Mogadishu) and Puntland regions where recent dengue outbreaks have been reported.

Recent Somali data show that dengue is no longer sporadic. In Banadir, 10.8% of 735 febrile patients were IgM-positive and 11.8% were NS1-positive, confirming active outbreak conditions across multiple districts [3]. In Puntland, 118 confirmed cases were identified among 956 suspected patients, with cases peaking in December 2022 and rising again in April–May 2023, a pattern the investigators linked to weather variability and rainfall [2]. These observations are epidemiologically important because rainfall and temperature shape mosquito proliferation and dengue incidence, yet Somalia still lacks a robust early warning architecture that integrates such signals prospectively [2].

Climate-informed forecasting is already technically feasible. A systematic review of 99 dengue prediction models found that all used climate predictors, 70.7% relied on climate variables alone, and machine learning was used in 39.4% of models; yet only 5.2% reported external validation [1]. This gap matters for Somalia, where models must be built for transferability, sparse data, and operational realism rather than retrospective accuracy alone.

Applied studies also show both promise and caution. In Vietnam, lagged climate variables improved dengue prediction, and a negative binomial model outperformed SARIMAX, XG Boost, and LSTM for weekly incidence forecasting [5]. However, LSTM and related models struggled to capture short-term outbreak peaks, which are precisely the events that matter most for emergency response [5]. Somalia therefore needs parsimonious, locally validated models that combine predictive performance with interpretability and resilience to missing data.

Google search data could strengthen this effort by compensating for delayed official reporting. In Brazil, models that incorporated Google Trends improved the accuracy of weekly dengue nowcasts across states compared with approaches based only on routine surveillance data [4]. Digital data are not a substitute for case confirmation, but they can provide real-time situational awareness and support earlier public communication and vector-control action [4].

For Somalia, the strongest argument is strategic rather than merely technical. Fragile health systems cannot afford to wait for laboratory-confirmed surges before acting. A localized forecasting framework that integrates district-level climate variables, historical case counts, and Somali-language or regionally relevant Google search trends could identify outbreak signals earlier, prioritize scarce diagnostics, and target interventions to high-risk districts. Without such innovation, recurrent dengue outbreaks will continue to be detected late, managed expensively, and controlled inequitably.

Establish district-level dengue surveillance that links meteorological, laboratory, and case-notification data streams [3,5].

Develop and externally validate hybrid forecasting models using climate lags, routine incidence, and Google Trends signals [1,4].

Invest in laboratory capacity and outbreak analytics so predictions can trigger rapid field verification and response [2,3].

Somalia should not remain absent from the emerging science of dengue early warning. Immediate investment in climate- and search-informed machine learning models is justified, feasible, and increasingly necessary to prevent localized outbreaks from becoming recurrent public health emergencies.

## Clinical trial

No applicable.

## Ethical approval

This study did not involve any human or animal subjects, and thus, did not require review by an Institutional Review Board (IRB).

## Funding

The authors have not been recipients of any funding for this specific study. Not applicable.

## CRediT authorship contribution statement

**Mohamed Mahdi Hashi:** Conceptualization, Writing – original draft. **Abdisamad Abdihakim Aadam:** Resources. **Abdishakur Ahmed Mahad:** Resources. **Abdimalik Dahir Ali:** Writing – original draft. **Yusuf Abdullahi Hubow:** Supervision.

## Declaration of competing interest

The authors declare that they have no known competing financial interests or personal relationships that could have appeared to influence the work reported in this paper.

## Data Availability

No datasets were generated or analyzed during the current study.
